# The Great Escape: the systems biology of endocrine resistance and lineage plasticity

**DOI:** 10.1530/ERC-26-0130

**Published:** 2026-08-27

**Authors:** Christo P C Dragnev, Zirui Fu, Sida Huang, Curtis J Perry, Adriana Kahn, Michaela A Dinan, Wei Shen Tan, Michael Leapman, David A Braun, Joshua Warrick, Himisha Beltran, Ping Mu

**Affiliations:** ^1^ Department of Urology, Yale School of Medicine, New Haven, Connecticut, USA; ^2^ Department of Chronic Disease Epidemiology, Yale School of Public Health, New Haven, Connecticut, USA; ^3^ Yale Cancer Center, Yale School of Medicine, New Haven, Connecticut, USA; ^4^ Department of Medical Oncology and Hematology, Yale School of Medicine, New Haven, Connecticut, USA; ^5^ Department of Pathology, Yale School of Medicine, New Haven, Connecticut, USA; ^6^ Department of Medical Oncology, Dana-Farber Cancer Institute, Boston, Massachusetts, USA

**Keywords:** lineage plasticity, endocrine resistance, prostate cancer, breast cancer, systems biology, single-cell transcriptomics, trajectory inference, multi-omics integration, neuroendocrine differentiation, and identity erosion

## Abstract

Endocrine therapies initially enforce lineage identity in hormone-driven cancers, yet sustained hormonal suppression often triggers a coordinated systems-level rewiring through lineage plasticity. Previously, lineage plasticity has been characterized as a discrete bypass mechanism, which lacks fluidic identity changes. Here, we describe the process as a progressive, adaptive trajectory of endocrine escape. We describe how the disruption of hormone receptor-anchored identity programs creates permissive conditions for transitions into HR-indifferent or neuroendocrine- or basal-like states. Next, we summarize historical methodologies for investigating lineage plasticity, such as patient-derived organoids, genetically engineered mouse models (GEMMS), lineage tracing, and single-cell multi-omics, and discuss novel systems biology approaches to enable the early detection and modeling of these unstable intermediate states. Finally, we present promising and potential uses for artificial intelligence and machine learning for dissecting therapy-induced identity shifts and resistance mechanisms. Intercepting these trajectories before lineage commitment offers a critical window for precision oncology interventions.

## Introduction

### Endocrine homeostasis, lineage stabilization, and the problem of escape

#### Hormone receptors as defining cell state

The survival and identity of most prostate (PCa) and breast cancers (BCa) are fundamentally hormone-dependent, relying on sex steroids such as androgens and estrogen to drive their respective oncogenic programs. These hormones bind to cytosolic nuclear receptors, such as the androgen receptor (AR) and estrogen receptor (ER), triggering receptor homodimerization and nuclear translocation. Once in the nucleus, these complexes function as master transcription factors, activating proliferative gene networks while simultaneously enforcing lineage identity. The dominance of these lineage-stabilizing programs is evidenced by the fact that nearly all untreated prostate cancers are AR-positive, while approximately 70% of breast cancer cases are ER-positive (1, 2). To counteract these drivers, next-generation endocrine therapies have been developed, including high-affinity AR antagonists like enzalutamide for PCa, and ER-targeted agents such as tamoxifen and the selective estrogen receptor degrader (SERD) fulvestrant for BCa. Other FDA-approved oral SERDs include elacestrant and imlunestrant to treat ER+ HER2-negative BCa. While these interventions are initially efficacious in suppressing tumor growth, chronic selective pressure frequently leads to recurrence, characterized by a transition to hormone-independent states.

#### Limits of classical therapy resistance paradigms

Traditionally, resistance to endocrine therapy has been viewed through the lens of hormone receptor target-centric modifications. In both PCa and BCa, the majority of tumors maintain a signaling reliance on their primary nuclear receptor (NR) driver through gene fusions, genomic amplification, point mutations in the ligand-binding domain, or the expression of constitutively active splice variants (3). However, the frequency and nature of these adaptations differ significantly between the two malignancies. In PCa, most of the enzalutamide-resistant cases exhibit AR overexpression driven by gene or enhancer amplification (4, 5), whereas ER amplification is rare in BCa, and loss of ER expression is the more prevalent resistant phenotype (6). These divergent evolutionary trajectories are likely influenced by the relative pharmacological selection pressures of the agents used. While tamoxifen binds ER with higher affinity to its natural ligand, estradiol (E2) (7), the antiandrogen enzalutamide possesses a significantly lower affinity for AR than dihydrotestosterone (DHT) (8). This weaker competitive blockade in PCa may favor the selection of clones that overexpress AR to restore canonical signaling, while the more robust blockade in BCa often drives the system toward a loss of hormonal reliance. In some PCa and BCa, mechanisms of resistance include loss of NR expression and a switch to alternative molecular drivers by integration of somatic and germline data by genomic and transcriptomic analysis of large patient cohorts (5, 9).

#### Lineage plasticity as adaptive endocrine escape

Emerging evidence frames the transition to hormone independence as a systems-level phenomenon called lineage plasticity. Now considered a hallmark of cancer, lineage plasticity is defined as acquired alteration of cell identity by de-differentiation, blocked differentiation, or trans-differentiation as a result of therapy selection pressure (10). The loss of hormone dependence is a critical consequence of lineage plasticity. In PCa, the trans-differentiation from a luminal/epithelial tumor to a neuroendocrine-like tumor results in resistance to androgen-deprivation therapy (11, 12). A parallel process occurs in BCa, where chronic ER suppression promotes epithelial-to-mesenchymal plasticity (EMP) and the expansion of the breast cancer stem cell (BCSC) pool (3, 13). These stem-like populations demonstrate acquisition of lineage plasticity, driving resistance to therapy, with estrogen propagating this population (13, 14, 15). Selection pressure from therapy after acquisition of lineage plasticity may allow for loss of reliance on estrogen in BCSCs, resulting in resistance to anti-estrogen therapies.

## Lineage plasticity: a conserved adaptive principle in hormone-driven cancers

Lineage plasticity is increasingly recognized as a unifying mechanism of therapeutic evasion across hormone-driven malignancies subjected to chronic endocrine pressure (16, 17, 18). Rather than an idiosyncratic, disease-specific phenomenon, plasticity reflects a conserved systems-level adaptation to the disruption of hormone receptor (HR)-anchored identity programs (16, 17, 19). This shift allows tumors to bypass the selective pressure of receptor inhibition by transitioning between cellular states (20).

### Prostate cancer: the prototypic model

Prostate cancer serves as the paradigm for endocrine-induced plasticity. The AR functions as a dominant lineage-stabilizing hub, orchestrating transcriptional programs that enforce luminal epithelial identity (21). While AR-targeted therapies initially induce growth arrest, sustained inhibition progressively destabilizes these lineage constraints (11, 12).

Clinically, this manifests as a spectrum of AR-indifferent states, including neuroendocrine prostate cancer (NEPC), hybrid luminal-basal phenotypes, and intermediate ‘amphicrine’ states (22, 23, 24). These transitions are not discrete switches; rather, they represent a trajectory of increasing transcriptional ‘entropy’. In this context, increasing transcriptional entropy reflects the progressive expansion of heterogeneous gene expression programs and the erosion of a dominant, AR-anchored lineage state, resulting in greater cell-state variability and regulatory permissiveness. This continuum challenges traditional binary classifications of AR-positive versus AR-negative disease, suggesting that resistance is a dynamic evolutionary process through a permissive regulatory landscape (17, 25).

### Breast cancer as a complementary context

Breast cancer offers a complementary endocrine context in which similar principles of lineage plasticity apply. Similar to AR in prostate epithelium, ERα signaling sustains luminal lineage identity by maintaining a hormone-dependent transcriptional circuitry in cooperation with lineage-determining factors such as FOXA1 and GATA3 (26, 27). Despite differences in tissue origin and receptor biology, breast and prostate cancers share striking parallels in their adaptive behaviors. In both contexts, hormone suppression precedes identity destabilization, and intermediate hybrid states emerge prior to the establishment of stable resistance (12, 28). These similarities reinforce the notion that endocrine escape is governed by a shared regulation rather than by receptor-specific mechanisms alone.

### General principles of endocrine-driven plasticity

Across hormone-driven cancers, several core principles recur that define the plastic response. Hormone receptors function as lineage constraints rather than growth signals, and their chronic suppression reshapes the regulatory landscape to create permissive conditions for lineage plasticity (21, 26). Viewed through this lens, endocrine resistance reflects a systems-level failure of identity maintenance. Understanding this failure requires approaches capable of resolving the gradual, coordinated rewiring that occurs across molecular layers over time. This complexity motivates the development of the systems biology frameworks discussed in the following sections.

## Foundational systems-level approaches: what they revealed and what they missed

### Classical approaches

**Figure 1:**
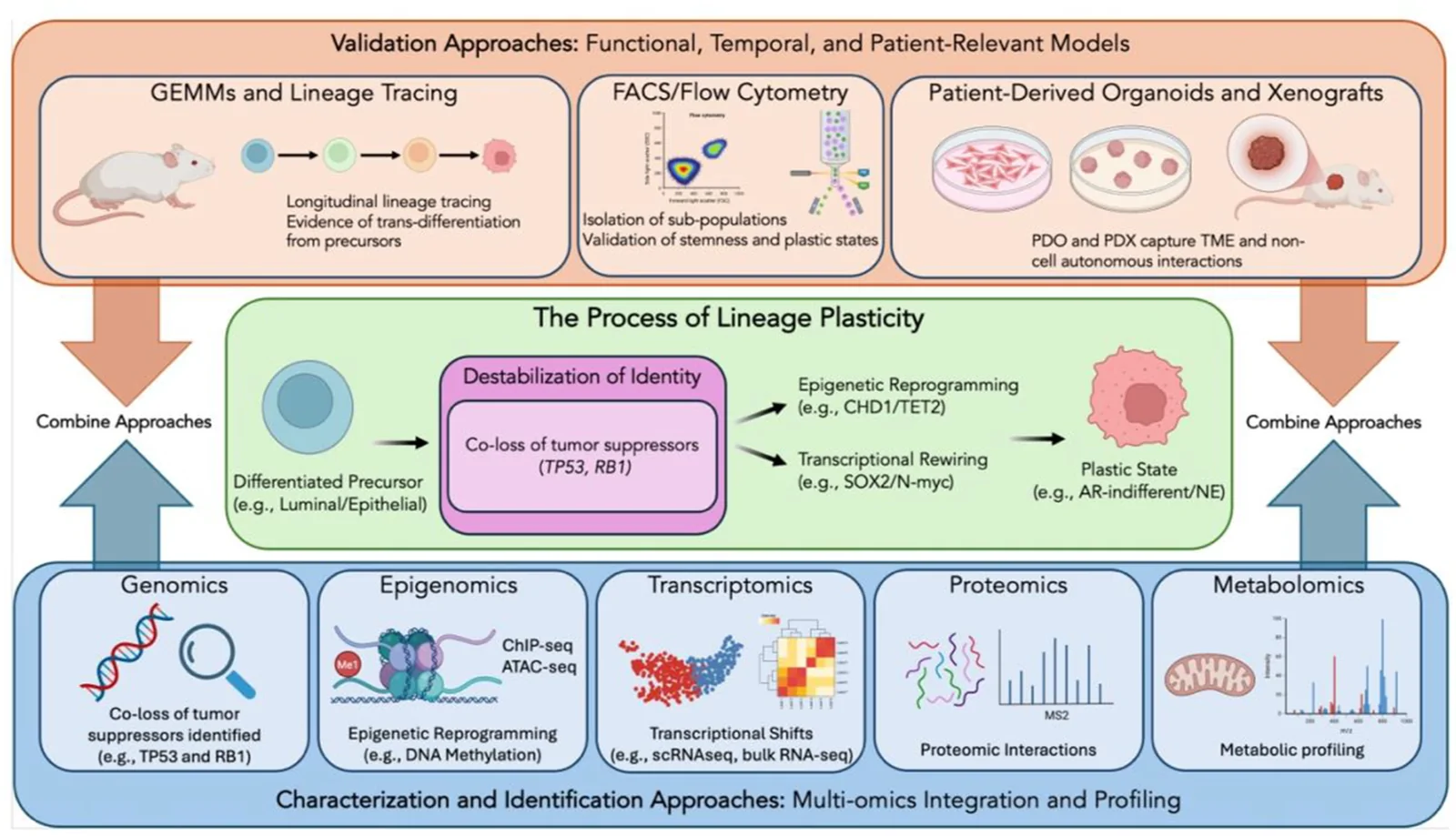
Systems-level interrogation of lineage plasticity across classical and multi-omic frameworks. (Top) Functional and patient-relevant validation approaches. GEMMs with lineage tracing provide temporal evidence of trans-differentiation from differentiated epithelial/luminal precursors. FACS/flow cytometry isolates and functionally interrogates plastic subpopulations. Patient-derived organoids and xenografts (PDOs/PDXs) model clinically relevant tumor–microenvironment interactions. (Bottom) Multi-omic characterization. Integrated genomics, epigenomics, transcriptomics, proteomics, and metabolomics define the molecular drivers and systems-level network remodeling associated with endocrine pressure. (Middle) Convergent process of lineage plasticity. Co-loss of tumor suppressors (TP53, RB1) destabilizes lineage identity, triggering epigenetic reprogramming and transcriptional rewiring (e.g. SOX2, N-myc), culminating in AR-indifferent or neuroendocrine-like plastic states. Figure generated using BioRender and Google Gemini.

Understanding lineage plasticity was established through the combination of genetically engineered mouse models (GEMMs), flow cytometry, cytology and histology, and bulk transcriptomic profiling (Fig. 1; Table 1). These systems-level platforms identified the co-loss of gatekeeper tumor suppressors, such as TP53 and RB1, as permissive that destabilize differentiated cell identity and allow for the adoption of alternative phenotypes (11, 12). Longitudinal lineage tracing in these models, utilizing fluorescent reporters or genetic barcodes, provided evidence that resistant variants arise directly from mature epithelial/luminal precursors through an adaptive process of trans-differentiation (11, 29). In addition, cellular tracking allows researchers to monitor clonal expansion of lineage plastic or stem-like populations, as well as the tissue it migrates to and its clonal evolution (11, 14, 30, 31). Complementing these methods, antibody-based tools like fluorescence-activated cell sorting (FACS) have been used to isolate distinct tumor subpopulations based on surface marker expression (32). Previous work has combined single-cell RNA sequencing (scRNA-seq) with FACS to compare transcriptionally identified clusters to surface marker-sorted subpopulations of cancer cells and validate predicted stemness, plasticity, and therapy-resistance capabilities (33). By quantifying markers associated with stemness, mixed-phenotypes, or EMP, researchers identified rare, therapy-resistant subpopulations.

**Table 1:** Summary of traditional and newer approaches to resolve systems-biology level interrogation of lineage plasticity, highlighting key questions asked, and corresponding strengths and weaknesses.

Approach category	Specific technologies	Biological questions	Strengths	Weaknesses/limitations
Traditional	Flow cytometry/FACS	How can distinct tumor subpopulations be isolated based on surface marker expression to study therapy resistance?	Identification and quantifies rare subpopulations Investigation of cell function in isolated subpopulations	Requires predefined surface markers Disrupts native tissue architecture Limited choice for cell defining features
	Bulk multi-omics (genomics, bulk RNA-seq, epigenomics, proteomics, metabolomics)	What are the dominant genetic alterations and broad transcriptional/epigenetic shifts driving hormone resistance?	Identification of molecular drivers and coupled epigenetic remodeling factors Establishes foundational networks of lineage plasticity Proteomics reveals activation of signaling pathways and post-translational modifications not captured at the RNA level Metabolomics identifies metabolic rewiring associated with therapy resistance	Signal averaging masks intratumoral heterogeneity and rare transitional states Provides static snapshots; fails to resolve the dynamic kinetic trajectory of escape Integration across multiple omics layers can be analytically complex
Newer	Single-cell transcriptomics (scRNA-seq and scATAC-seq, trajectory inference)	What are the continuous, transitional, or hybrid states cells navigate as they evade hormone dependence?	Examination of cell state continuum and rare hybrid phenotypes Modeling pseudo-time models directional evolutionary paths scATAC-seq identifies chromatin accessibility landscapes and transcription factor regulatory programs underlying lineage transitions Integration of scRNA-seq and scATAC-seq links transcriptional states to epigenetic reprogramming during endocrine escape	High-dimensional data requires complex harmonization and simplification Inference of dynamic trajectories from static single-cell snapshots Multi-omic integration (RNA + chromatin) increases computational complexity and batch effects
	Spatial transcriptomics	How do local spatial gradients create micro-niches that favor lineage plasticity?	Definition of cell types in the TME Identification of context-dependent and non-cell-autonomous signals	Limited to the specific biopsy/tissue slice
	AI and computational systems modeling	Can we predict future resistance trajectories and model cellular instability by integrating diverse data types?	Unification of large multi-modal data Predication of prognostic factors and therapy response	Requirement of strict biological interpretability and validation

### Key insights gained through classical approaches

Foundational systems-level interrogation, primarily through GEMMs and bulk-level molecular profiling, redefined therapy resistance as a coordinated network shift rather than a series of isolated genetic failures. These classical approaches substantiated the multi-step process allowing for therapy escape, in which the co-loss of gatekeeper tumor suppressors destabilizes cell identity through epigenetic reprogramming. Complementing these methods, cell-sorting and flow cytometry technologies enabled the identification and isolation of rare, resistant subpopulations for targeted functional investigation. This destabilized environment permits genetic regulators, such as SOX2 or N-myc, to actively reprogram the cellular regulatory network toward hormone receptor-indifferent phenotypes. Longitudinal lineage tracing studies in these foundational models provided proof that these resistant states arise directly from epithelial/luminal precursor populations through trans-differentiation (34). Finally, early pharmacological perturbation experiments demonstrated that this systems-level rewiring is potentially reversible, as targeting epigenetic hubs can force re-differentiation to restore lineage identity and sensitivity.

### Core limitations to classical approaches

**Figure 2:**
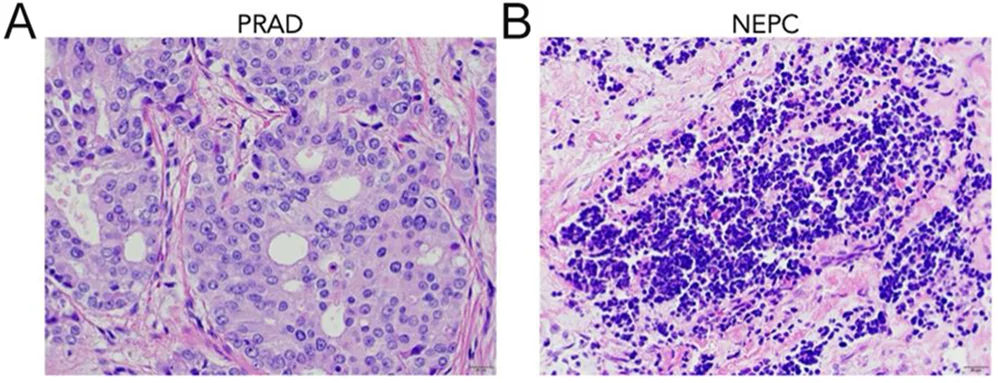
Two main histological subtypes of castration-resistant prostate cancer in histological transformation. A. prostate adenocarcinoma (PRAD) and B. small cell/neuroendocrine prostate cancer (NEPC). Scale bars, 20 μm.

Despite their foundational role, classical systems-level analyses are constrained by significant technological and biological barriers. Bulk-level profiling relies on signal averaging, which effectively masks the diverse intratumoral heterogeneity and rare transitional states that define the early stages of endocrine escape. These models often provide static snapshots of tumor evolution, failing to resolve the temporal and spatial kinetics and dynamics of therapy resistance. A biological limitation is the frequent reliance on immunodeficient xenograft models, which neglect the complex immunological and stromal interactions within the tumor microenvironment (TME). Emerging evidence suggests that full histological transformation, such as the transition from prostate adenocarcinoma (PRAD) to neuroendocrine PCa (NEPC) (Fig. 2), often requires non-cell-autonomous signals that are absent in conventional *in vitro* or orthotopic models (29). Finally, species-specific differences in immune signaling and technical hurdles in GEMMs, such as promoter-dependent temporal Cre activation, underscore the need for high-resolution, patient-relevant platforms capable of capturing the dynamic identity shifts of cancer.

## Multi-omics integration reveals regulatory rewiring for endocrine pressure escape

### Coupled transcriptional and epigenetic remodeling

While genomic loss of tumor suppressors, such as *TP53* and *RB1*, is a necessary groundwork for lineage plasticity and therapy resistance, the functional transition is driven by a global transcriptional and epigenetic shift that allows tumor cells to navigate a spectrum of cell identities (11, 12, 31, 35). Development-related transcription factors (TFs) have been shown to regulate lineage plasticity, specifically lineage-specific TFs that are typically silenced in differentiated cells but activated in tumors (10, 35). In addition, key epigenetic processes, such as post-translational modification through histone proteins and DNA methylation, have been directly linked to regulating lineage plasticity in cancer (35, 36, 37). In breast cancer, these observations are already beginning to translate into epigenetic therapeutic strategies. In the first-in-human phase 1 study of the selective KAT6A/B inhibitor PF-07248144 in heavily pretreated ER+ HER2-negative metastatic breast cancer, the PF-07248144-fulvestrant combination achieved an objective response rate of 30.2% and a median progression-free survival of 10.7 months (38). This has renewed interest in induced-proximity strategies such as RIPTACs, a heterobifunctional ‘hold-and-kill’ platform designed to use tumor-selective target expression to disable an essential cofactor and thereby broaden the therapeutic window. Consistent with this translational momentum, Pfizer has advanced PF-07248144 toward phase 3 testing, while additional KAT6-directed programs from Menarini/Stemline (MEN2312) and BeOne (BG-75098) are also in development for HR+/HER2-negative breast cancer. In parallel, Halda Therapeutics is developing a breast-cancer RIPTAC program for hormone receptor-positive metastatic disease, further supporting the therapeutic potential of ER/BRD4-directed induced-proximity approaches in overcoming endocrine escape. In endometrial cancers, machine learning and multi-omics analysis revealed how alterations to 3D-genomic structures can affect tumorigenesis (39). Integrated multi-omics and spatial experiments have been used to identify EMP and trans-differentiation processes as a result of transcriptional and epigenetic changes (35). Newer approaches, such as using cell-free DNA (cfDNA) methylation, cfDNA nucleosome footprinting, and 5-hydroxy-methylcytosine (5hmC) sequencing, allow for detecting transcriptional and epigenetic changes linked to lineage plasticity, providing a new method for using liquid biopsies for lineage plasticity tracking (31, 35). Metabolic profiling has been an understudied aspect of lineage plasticity, but transcriptomic and epigenetic profiling through RNA-seq, ATAC-seq, and ChIP-seq have validated how metabolic shifts drive epigenetics required for lineage switching (Fig. 1) (40). Together, these coupled molecular remodeling approaches monitor shifting cellular identities and characterize vulnerabilities within the spectrum of hybrid cancer cell states.

### Proteomic and post-transcriptional layers

Proteomic and post-transcriptional layers serve as an essential complement to transcriptional and epigenetic profiling for studying functional regulators of lineage plasticity. Integrative analyses of multi-omics have been used to determine drivers of lineage plasticity in prostate cancer and identify hybrid states (41, 42). Emerging systems-level frameworks, like NuRome, move beyond descriptive -omics technology by integrating high-dimensional cistrome and transcriptome data with proteomic interactomes to predict how nuclear receptors directly dictate cell fate transitions (43). Spatial proteomics has recently been used in combination with traditional genomics approaches to characterize breast cancer heterogeneity and identify components of the tumor microenvironment (TME) that shape cellular plasticity (44). Proteomic and transcriptomic data should be analyzed in parallel, as differences in transcript and protein levels can reveal alternative post-translational regulatory mechanisms (45). In addition, functional resolution of epigenetic-driven lineage plasticity regulatory programs can be explored through single-cell proteomic technologies, such as epigenetic-focused CyTOF to measure the wide spectrum of histone modifications on single cells from tumors (46). By integrating these layers into a multi-omic atlas, systems biology frameworks can model resistance as a measurable and predictable trajectory of network stabilization.

## Single-cell and spatial approaches: resolving transitional and hybrid states

**Figure 3:**
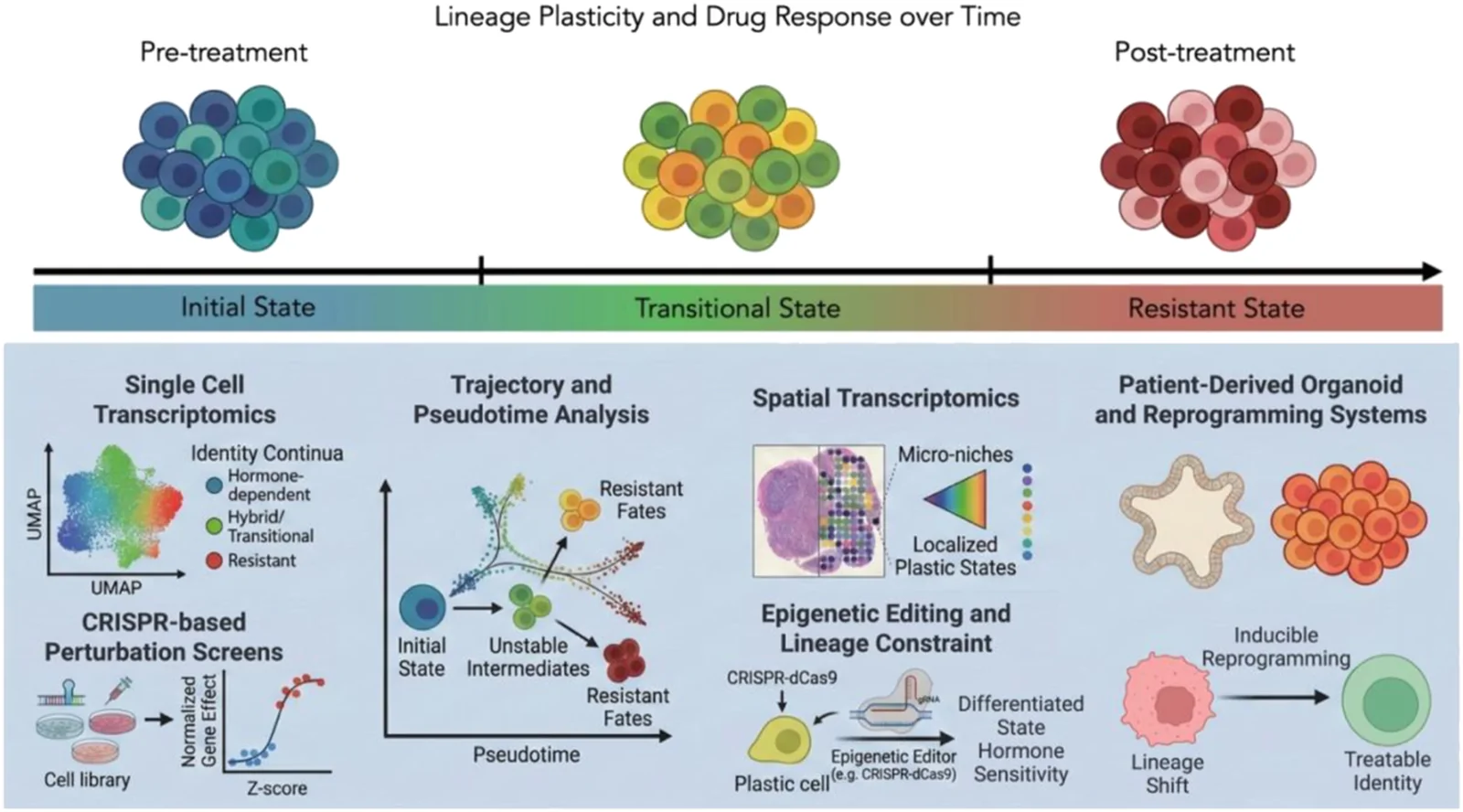
High-resolution and functional approaches to deconstruct lineage plasticity. Tumor cell progression from a pre-treatment hormone-dependent state through heterogeneous transitional states toward post-treatment resistance is a continuous process. Single-cell transcriptomics and trajectory analyses reveal a spectrum of cell states that transition through unstable intermediates toward resistant fates. Spatial transcriptomics maps localized plastic niches within the tissue microenvironment. Functional approaches include CRISPR-based perturbation screens to identify essential plasticity regulators and epigenetic editing strategies designed to restore lineage constraint and enforce re-differentiation into treatable states. Patient-derived organoids capture intrinsic plasticity, while inducible reprogramming systems allow for forced lineage shifts to establish causal regulatory links. Figure was generated using Google Gemini.

The emergence of lineage plasticity long predated its formal detection. Historically, traditional bulk sequencing measurements obscured transitional states through signal averaging, creating a deceptive illusion of abrupt, binary phenotypic shifts (47, 48). Modern single-cell and spatial technologies have since revealed that this plasticity was previously unresolved rather than absent, providing a high-resolution view of the cellular maneuvers that define endocrine escape (Fig. 3; Table 1) (28, 49).

### Single-cell transcriptomics and identity continua

Single-cell transcriptomic profiling has fundamentally reframed endocrine resistance as a continuous, rather than categorical, process. Besides observing discrete cell types, researchers now recognize that hormone-treated tumors contain heterogeneous populations distributed along fluid identity continua. These cells frequently exhibit partial hormone receptor activity, mixed lineage markers, and significantly elevated transcriptional heterogeneity (26, 49, 50, 51, 52). This heterogeneity reflects increased cell-to-cell variability in gene expression and the simultaneous activation of lineage-defining programs that are normally mutually exclusive. Such co-expression patterns indicate a loosening of lineage constraints and the emergence of unstable, hybrid cellular states.

These hybrid phenotypes are particularly enriched under chronic endocrine pressure, where cells retain fragments of their original identity while simultaneously activating alternative regulatory programs. Crucially, such states often precede overt clinical resistance and may persist at low frequencies long before progression is detectable. Longitudinal single-cell analyses suggest that these rare intermediate populations expand under continued therapeutic selection, ultimately contributing to the emergence of stable resistant clones. This observation highlights plasticity as an early adaptive response rather than a late-stage anomaly, suggesting that the ‘seeds’ of resistance are sown early in the treatment course (49, 53, 54).

### Trajectory inference and pseudotime modeling

The application of trajectory inference methods has enabled the reconstruction of complex endocrine escape paths from static single-cell snapshots (55). Rather than depicting resistance as a binary switch, pseudotime analyses reveal a directional progression in which hormone-dependent cells transition through transcriptionally unstable intermediate states before diverging into distinct resistant phenotypes. These intermediate states are characterized by increased variability in lineage-associated gene expression and partial activation of alternative regulatory programs, consistent with weakened lineage constraint. These trajectories are rarely linear; instead, they display bifurcations, convergence points, and reversible segments that reflect the underlying regulatory instability imposed by therapeutic intervention (55, 56). Such models challenge deterministic views of resistance. Cells do not inexorably march toward a single, predefined resistant endpoint. Instead, they explore a constrained ‘state space’ shaped by the intensity of therapy, the specific genetic context of the clone, and varying microenvironmental cues (31, 35, 57). Here, state space refers to the spectrum of transcriptionally accessible cellular identities permitted by the evolving regulatory landscape. This stochastic exploration suggests that resistance is a probabilistic outcome of cellular instability (55, 57).

### Spatial transcriptomics and microenvironmental context

Spatial profiling adds a critical dimension by situating plastic transitions within the native tissue architecture. Research has shown that hormone signaling, lineage identity, and cellular stress responses are often spatially uncoupled, producing localized micro-niches where plastic states preferentially emerge (57, 58). These niches often correspond to gradients of drug exposure, specific stromal interactions, hypoxia, or paracrine immune signaling. Spatial analyses reveal that plasticity is not a cell-intrinsic property alone but is deeply context-dependent (58, 59). Collectively, these findings support a model in which permissive niches stabilize hybrid identities, potentially biasing the probability of transition toward more durable resistant states. This spatial heterogeneity underscores the importance of local constraints in shaping the ultimate trajectory of endocrine escape (57, 58, 59).

### Clinical implications of high-resolution mapping

The ability to resolve transitional and hybrid states has profound clinical implications for the future of precision oncology. Detecting lineage plasticity before it culminates in overt resistance opens a critical window for early intervention. This capability potentially allows for therapeutic redirection or ‘adaptive’ dosing strategies designed to collapse unstable states before lineage commitment occurs (49, 53, 54). Consequently, single-cell and spatial approaches shift the clinical focus from identifying terminal resistant endpoints to intercepting the unstable trajectories currently in progress.

## Functional and reprogramming-based approaches for lineage tracking

### CRISPR-based perturbation screens

CRISPR-based perturbation models serve as a dominant systems biology tool for identifying specific genes and testing causal necessity and sufficiency within regulatory networks in both *in vitro* and *in vivo* settings. By using lineage tracking systems that exploit CRISPR technology, researchers can construct high-resolution phylodynamic mapping of cellular evolutionary trajectories as cells transition between cell states (Fig. 3). Focused CRISPR interference/knockout screening in combination with multi-omics, such as with the NuRome pipeline (43), incorporates genetic perturbations with the multi-ome to test how specific enhancers, activators, or receptors act as controllers of plastic identities. These functional genetic approaches are particularly effective at investigating infrequently mutated genes, as demonstrated through a CRISPR-Cas mutagenesis screen in BCa revealing the diverse role of epigenetic regulators (EpiDrivers) on tumorigenesis (60). In addition, integrating multi-omics, like ATAC-seq and RNA-seq, with CRISPR-based screens and functional validation assays provided evidence that loss of *CHD1*, a chromatin remodeler, contributes to antiandrogen therapy resistance through chromatin dysregulation by a diverse array of transcription factors (61). CRISPR-based screens transform static molecular models into dynamic, targetable systems to investigate therapy escape by resolving which rewired cellular components are essential for maintaining the plastic state.

### Epigenetic editing and lineage constraint

Epigenetic editing and differentiation-based systems represent transformative approaches to restore lineage constraint and enforce differentiated cell identity in plastic cancers. These reprogramming-based techniques aim to override plastic tumor therapy evasion by targeting hubs that maintain undifferentiated or alternative states, most notably EZH2, LSD1, and SOX2, whose inhibition can force re-differentiation back into hormone-sensitive luminal lineages (11, 12, 35, 62, 63). Recent work on epigenomic remodeling has demonstrated that CRISPR-mediated or pharmacological targeting of the histone methyltransferase NSD2 can durably revert neuroendocrine variants into adenocarcinoma phenotypes, successfully restoring canonical receptor programs and drug sensitivity (36). Similarly, investigating the molecular switch between lineage coactivators, such as the Zinc-Finger protein ZNF397, and plastic modifiers, such as TET2, has revealed that inhibiting epigenetic mediators of 5hmC rewiring can selectively prevent multi-lineage potential and resensitize tumors to endocrine pressure (64). This strategy of enforced trans-differentiation leverages the intrinsic reversibility of the epigenetics to lock plastic cells into stable, treatable identities.

### Patient-derived organoid and reprogramming systems

Patient-derived organoids (PDOs) offer controlled and flexible platforms for modeling lineage plasticity under defined selective pressures (29, 65, 66, 67, 68). Unlike traditional two-dimensional cell lines, organoids preserve the three-dimensional structure and intrinsic plasticity of the original tumor. Extensive efforts have established PDO biobanks that serve as high-fidelity surrogates, representing the clinical heterogeneity of patient tumors, including diverse hormone statuses, mutational profiles, and variable treatment responses (66, 67, 68). To capture spatial and temporal dynamics, PDOs are frequently integrated with *in vivo* models. Patient tumors are routinely engrafted into immunocompromised mice to create patient-derived xenografts (PDXs), which can be subsequently extracted and cultured *ex vivo* as matched organoids (69). Consequently, previous work used *ex vivo* organoids and xenografted them back into mice to monitor tumor evolution and track plasticity. Coupled with fluorescent markers, this pipeline allowed for the *in vivo* spatial mapping of cell migration, metastasis, and lineage plasticity (70).

Reprogramming-based systems enable the induction or reversal of specific lineage states. Using inducible transcription factors or CRISPR-based editing, researchers can establish causal links between regulatory rewiring and phenotypic outcomes. Specifically, previous work demonstrated that primary human tumor organoids can be phenotypically and functionally altered through CRISPR-Cas9 genome editing to reprogram tumorigenesis (71). In addition, recent advancements have used CRISPR screens within PDOs to elucidate complex drug–gene interactions and identity potential mechanisms of resistance (72). To streamline the process, another work has developed a pipeline to culture organoids from normal breast and breast tumor tissue, perform genetic manipulation, and orthotopically xenograft tumors into mice within 4 weeks (73).

By leveraging patient genomic data, these approaches can evaluate inter- and intratumor heterogeneity. Together, they can be used to pinpoint patient-specific shifts in cellular identity, allowing for the ‘forced’ exploration of the survival fitness landscape to identify the molecular thresholds for lineage shifts. Thus, organoids serve as an attractive, reliable model for bridging abstract mechanistic insights with translational relevance, anchoring systems-level observations in experimentally tractable and testable frameworks.

## Computational and AI-based systems modeling: predicting the escape trajectory

The recognition of endocrine resistance as a dynamic trajectory rather than a discrete endpoint naturally motivates computational frameworks capable of modeling continuous state transitions (55, 74, 75). While single-cell and spatial technologies resolve identity heterogeneity at high resolution, computational and AI-based systems modeling provide the means to integrate these observations into predictive representations of lineage instability under endocrine pressure (47, 55, 74).

### Latent state modeling of identity instability

A central conceptual advance in computational modeling of lineage plasticity is the treatment of cell identity as a continuous latent state space rather than a collection of discrete phenotypes. Single-cell transcriptomics generates high-dimensional data where each cell’s identity is defined by thousands of gene expression variables. Recently, manifold learning has been used to compress this complex data into a lower-dimensional latent space, denoising the data and mathematically mapping phenotypic identity instability (76). In this framework, hormone receptor-anchored lineages occupy relatively stable basins, while endocrine therapy reshapes the cellular landscape, flattening lineage constraints and permitting exploration of intermediate, unstable states (77, 78). Latent variable models provide a principled way to capture this instability, representing plasticity as increased variance and entropy within identity manifolds, rather than discrete cell state switches (79).

To precisely characterize this continuum, probabilistic models, such as deconvolution of spatial transcriptomic profiles using variational inference (DestVI), are deployed to identify intra-cell-type variation and map continuous transcriptional states (80). Moreover, the random matrix theory algorithm and Leiden algorithm are used to detect rare cell populations, embed high-dimensional data into latent spaces, and project data for trajectory analysis in both human and mouse prostate cancer models (81).

Mapping these dynamics requires tools capable of capturing the multi-directional cell state changes inherent to cancer lineage plasticity. Standard pseudotime analysis typically enforces a probabilistic unidirectional map of gene expression, which often fails to capture the complex evolutionary routes of tumor cells. RNA velocity has emerged as an alternative to describe multi-directional dynamics through the ratio of spliced to unspliced RNA transcripts (76). However, its utility in cancer biology research remains limited due to batch effects, high genetic instability, and the wide variation in splicing rates exhibited by tumor cells. Thus, these advanced computational representations not only provide descriptive mapping but also capture the increased regulatory permissiveness imposed by endocrine suppression. The perspective of a cell state continuum directly aligns with experimental observations that hybrid and transitional states are a requisite precursor to durable therapy resistance (29, 49, 82).

### Trajectory forecasting under endocrine pressure

Beyond reconstructing past transitions, computational models increasingly aim to forecast future resistance trajectories. By learning directional trends within latent identity spaces, trajectory-based modeling enables the estimation of likely evolutionary paths under continued hormone suppression (83). Mathematical models are being increasingly in tested in the clinic to improve therapy responses (84, 85), such as tracking evolutionary paths of therapy-resistant cells and adjusting hormone therapy in metastatic castration-resistant prostate cancer (86). Another work used differential equation-based models to describe evolutionary dynamics of cell cycle transitions in endocrine therapy-resistant breast cancer (87). These approaches conceptualize resistance as a probabilistic outcome shaped by regulatory instability, genetic context, and microenvironmental constraints. One model, tradeSeq, utilizes single-cell RNA-seq to create a trajectory-based differential expression analysis of cell lineage (56), thus expanding the number of trajectory interference models currently available (55). Such forecasting reframes endocrine resistance as a predictable process rather than an inevitable deviation (55, 56). Cells do not uniformly converge on a single resistant fate; instead, they traverse constrained trajectories with identifiable branch points, suggesting that early deviations may carry prognostic and therapeutic significance.

### Multi-modal data integration

A defining strength of AI-based systems modeling lies in the capacity to integrate heterogeneous data types into coherent representations (74, 88, 89). Histology, transcriptomics, epigenomics, proteomics, and spatial context each capture distinct facets of lineage regulation, yet no single modality is sufficient in isolation. Multi-modal integration enables the alignment of molecular state, tissue architecture, and phenotypic behavior within unified latent frameworks (74, 88). Conceptually, these integrated representations allow lineage plasticity to be modeled as a systems-level property emerging from coordinated changes across regulatory layers (89). Rather than privileging any single data source, such frameworks emphasize relational structure and cross-modal coherence as defining features of endocrine escape.

### Interpretability and biological insight

While increasingly powerful, computational models must retain biological interpretability to remain relevant for translational oncology. Avoiding purely black-box predictions is particularly critical in the context of lineage plasticity, where mechanistic understanding guides therapeutic intervention (90, 91). Interpretability-focused modeling prioritizes features that map onto known regulatory programs, transcriptional networks, and spatial niches (91, 92). By preserving mechanistic relevance, computational systems modeling functions not only as a predictive engine but also as a hypothesis-generating tool, revealing organizing principles that govern identity destabilization under endocrine pressure (90, 91, 92).

## Integration, challenges, and future directions

The convergence of experimental and computational approaches has transformed the study of endocrine resistance, yet it has also highlighted fundamental challenges that no single method can resolve in isolation. Addressing lineage plasticity requires integrative frameworks that reconcile biological complexity with translational feasibility.

### Why no single approach is sufficient

Experimental systems excel at uncovering causal mechanisms but are inherently limited in scale and temporal resolution. Conversely, computational models can synthesize high-dimensional data but depend on biological grounding to avoid abstraction divorced from reality. The study of endocrine escape, therefore, necessitates complementarity across experimental perturbation, high-resolution profiling, and systems-level modeling. This integration ensures that computational predictions remain biologically anchored, while experimental findings are interpreted within a broader systems context.

### Technical and conceptual challenges

Several challenges continue to constrain progress. Data harmonization across platforms, modalities, and cohorts remains a major technical barrier, particularly when integrating spatial and temporal information. Temporal sampling constraints further complicate inference, as clinical specimens rarely capture the full evolutionary course of resistance. Translating complex systems-level insights into clinically actionable strategies also requires simplification without loss of biological fidelity. Overcoming these challenges will require coordinated experimental design, standardized data representations, and continued methodological innovation.

### Targeting lineage plasticity as a vulnerability

Viewing endocrine resistance as a trajectory opens new therapeutic opportunities. Rather than targeting terminal resistant states, interventions may be designed to intercept unstable intermediates, restore lineage constraint, or redirect cells toward less aggressive fates. Rational combination therapies that stabilize identity programs while suppressing alternative drivers may collapse permissive state spaces before commitment occurs. This strategy reframes plasticity not as an insurmountable obstacle, but as a transient vulnerability.

### Concluding perspective

Collectively, the evidence reviewed here supports a unifying view of endocrine resistance as a dynamic, systems-level process. Lineage plasticity represents an adaptive trajectory shaped by regulatory instability rather than a binary outcome. Systems biology approaches, spanning experimental, computational, and AI-based frameworks, now enable observation, prediction, and potentially redirection of this process. By integrating mechanistic insight with predictive modeling, the field moves closer to a proactive paradigm in which endocrine escape is anticipated and intercepted, rather than retrospectively treated.

## Declaration of interest

DAB reports share options in Elephas; advisory board, consulting, or personal fees from Cancer Expert Now, Adnovate Strategies, MDedge, CancerNetwork, Catenion, OncLive, Cello Health BioConsulting, PWW Consulting, Haymarket Medical Network, Aptitude Health, ASCO Post and Harborside, Targeted Oncology, Merck, Pfizer, MedScape, Accolade 2nd MD, DLA Piper, Dechert, AbbVie, Compugen, Link Cell Therapies, Scholar Rock, NeoMorph, Nimbus, Exelixis, AVEO, Eisai, Daiichi Sankyo, Caris, Ottimo Pharma, and Elephas; and research support from Exelixis and AstraZeneca, outside of the submitted work. MSL is associated with Blue Earth Diagnostics, Medtronic Inc, UpToDate. No other authors have COI to disclose.

## Funding

This work was supported or partially supported by: National Cancer Institute/National Institutes of Health: R37CA258730, R01CA28882, R01CA292949 to P. Mu; Prostate Cancer Foundation: 25CHAL05, 17YOUN12 to P. Mu, Yale Cancer Center CCSG Pilot Grant P30CA016359 to P Mu. Z Fu is supported by National Science Foundation Graduate Research Fellowship under Grant No. DGE-2139841.

## Author contribution statement

CPCD and ZF were involved in conceptualization, visualization, and writing of the original draft of this article. SH, CJP, MAD, WST, ML, and DB edited this manuscript. JW and HB visualized this study. PM supervised, as well as wrote, reviewed, and edited the article.

## Declaration of AI-assisted technologies in the manuscript preparation process

During the preparation of this work, the author(s) used ChatGPT to assist with proofreading and grammar correction. After using this tool, the author(s) reviewed and edited the content as needed and take full responsibility for the content of the published article. Google Gemini was used in a limited capacity to assist with the visual refinement of several figures. The original figure concepts, scientific content, and graphical elements were created by the author(s). Gemini was used only to help polish the layout and presentation. All figures were carefully reviewed and finalized by the author(s), who take full responsibility for their accuracy and content.

## Ethical approval and consent to participate

Not applicable. This review article does not report any new studies involving human participants, human specimens, animals, or identifiable patient data performed by the authors; therefore, ethics committee or IRB approval was not required.
